# Report from the World Health Organization's immunization and vaccines-related implementation research advisory committee (IVIR-AC) meeting, virtual gathering, 10–13 September 2024

**DOI:** 10.1016/j.vaccine.2024.126519

**Published:** 2025-01-01

**Authors:** Philipp Lambach, Sheetal Silal, Alyssa N. Sbarra, Mitsuki Koh, Rakesh Aggarwal, Habib Hasan Farooqui, Stefan Flasche, Alexandra B. Hogan, Sun-Young Kim, Kathy Leung, William J. Moss, Patrick K. Munywoki, Allison Portnoy, Meru Sheel, Xuan-Yi Wang

**Affiliations:** a Immunizations, Vaccines and Biologicals, World Health Organization, Geneva, Switzerland; b Modelling and Simulation Hub, Africa, University of Cape Town, Cape Town, South Africa; c Centre for Global Health, Nuffield Department of Medicine, Oxford University, Oxford, United Kingdom; d Johns Hopkins Bloomberg School of Public Health, Baltimore, MD, United States; e Jawaharlal Institute of Postgraduate Medical Education & Research, Puducherry, India; f College of Medicine, QU Health, Qatar University, Qatar; g Centre for Global Health, Charite, Berlin, Germany; h School of Population Health, University of New South Wales, Sydney, Australia; i Seoul National University, Seoul, South Korea; j School of Public Health, The University of Hong Kong, Hong Kong, SAR, China; k Kenya Medical Research Institute, Centre for Global Health Research, Nairobi, Kenya; l Department of Global Health, Boston University School of Public Health, Boston, United States; m Center for Health Decision Science, Harvard T.H. Chan School of Public Health, Boston, United States; n School of Public Health, Faculty of Medicine and Health, University of Sydney, Sydney, Australia; o Sydney Infectious Diseases Institute, Faculty of Medicine and Health, University of Sydney, Sydney, Australia; p Shanghai Institute of Infectious Disease and Biosecurity, Fudan University, Shanghai, China

**Keywords:** IVIR-AC, Vaccine, Immunization, WHO

## Abstract

The Immunization and Vaccines-related Implementation Research Advisory Committee (IVIR-AC) is the primary advisory body of the World Health Organization conducting independent reviews of immunization-related implementation research, with a primary focus on transmission and economic modeling analyses that estimate the value and impact of vaccines. From 10 to 13th September 2024, IVIR-AC convened virtually for its second of two semi-annual meetings to provide feedback and recommendations across six sessions including: pneumococcal vaccination strategies that rely on indirect protection; vaccine impact modeling for chikungunya; The Lancet Commission on strengthening the use of epidemiological modeling of emerging and pandemic infectious diseases; methods for immunization coverage estimation; setting immunization research priorities in the South-East Asian Region; and modeling evidence related to typhoid conjugate vaccine schedules. This report summarizes the sessions, proceedings, and recommendations from that meeting.

## 1 Context

The foundation of immunization programs and policy should be based in evidence. The Immunization and Vaccines-related Implementation Research Advisory Committee (IVIR-AC) serves to evaluate and review vaccine effectiveness and impact studies, value assessments, and modeling analyses related to priority topics as an advisory body to the World Health Organization (WHO) Immunization, Vaccines, and Biologicals (IVB) Department and contributing to the Strategic Advisory Group of Experts on Immunization (SAGE) evidence. During semi-annual meetings, IVIR-AC’s main role is to provide advice and recommendations to WHO on topic-based sessions; IVIR-AC has no executive or decision-making power [1]. This report summarizes proceedings and recommendations of IVIR-AC’s virtual meeting held from 10 – 13^th^ September 2024 [2].

## 2 Scope of the meeting

WHO IVB and SAGE requested six topics to be reviewed including: pneumococcal vaccination strategies that rely on indirect protection; vaccine impact modeling for chikungunya; The Lancet Commission on strengthening the use of epidemiological modeling of emerging and pandemic infectious diseases; methods for immunization coverage estimation; setting immunization research priorities in the South-East Asian Region; and modeling evidence related to typhoid conjugate vaccine schedules. For each session, background, objectives, and IVIR-AC feedback and recommendations are described below.

## 3 Summary of sessions

### 3.1 Session 1: Pneumococcal vaccination strategies that rely on indirect protection

Since their introduction in 2007, pneumococcal conjugate vaccines have contributed to substantial declines in incidence of pneumococcal disease in infants (i.e., direct and indirect effects of immunization) as well as in other age groups (i.e., indirect effects). In 2019, WHO set current recommendations that infants should receive either three primary doses without a booster (i.e., 3p + 0) or two primary doses with a booster (i.e., 2p + 1) [3]. However, at least seven clinical trials, in various stages and using different designs, suggest that reduced dose schedules (i.e., 1p + 1) could provide similar protection after boosting and thus sustain herd protection. Further, single-dose campaigns in high-risk settings (e.g., humanitarian emergencies) or different modalities of administration (e.g., fractional doses) may allow immunization programs to reduce the number of injections and costs while increasing programmatic feasibility. The WHO SAGE working group [4] on pneumococcal vaccines has been tasked to re-evaluate pneumococcal conjugate vaccination strategies for children, with recommendations to SAGE planned in March 2025. Their deliberations include: (i) whether available evidence, including from Phase IV trials, supports the use of two-dose (i.e., 1p + 1) PCV schedules and under which conditions; (ii) whether and under what circumstances evidence supports the use of fractional dosing; and (iii) the optimal age range and dosing for PCV multi-age cohort campaigns delivered in the context of humanitarian crises or other settings where routine coverage is very low. IVIR-AC was asked to comment on the methodology of available modeling analyses using data from clinical observational studies, whether their results were robust to answer the questions posed by the working group, and if the presented work plan was sufficient to generate additional key evidence prior to the SAGE meeting.

During the session, investigators from the London School of Hygiene and Tropical Medicine presented modeling work to assess the impact of alternative PCV dosing strategies. An age-structured compartmental model accounting for age-group specific transmission rates, both vaccine-type and non-vaccine-type serotype carriage, and case-carrier ratios to assess invasive pneumococcal disease (IPD) was used across studies to infer vaccine efficacy against carriage and disease and duration of protection. This model structure allows for the exploration of a range of dosing strategies, including different vaccine efficacy and waning rates.

IVIR-AC agrees that the modeling methodology presented is appropriate to provide evidence to the SAGE working group deliberations on the three streams of work encompassing reduced-dose routine PCV schedules, fractional dosing, and the use of PCV in humanitarian crisis settings. IVIR-AC notes that similar underlying model structures and parameterizations were used to explore these different modeling questions across work streams.

Using data from a cluster randomized trial in Nha Trang, Vietnam, a setting with low transmission and high vaccine coverage, modeled estimates suggest no reduction in infant vaccine-type prevalence and IPD impact from switching from a 2p + 1 to 1p + 1 schedule. Applying this model to settings with different transmission intensities, the following estimates have been generated. In the Gambia, a high transmission and high vaccine coverage setting where a cluster randomized trial assessing differences between 3p + 0 and 1p + 1 schedules is ongoing, model extrapolations suggest a 18 % (95 % CI: 9 to 34 %) increase in infant IPD impact when switching from a 3p + 0 to a 1p + 1 schedule. In Fiji, a setting with low transmission and high vaccine coverage, the model extrapolates that a schedule switch from 3p + 0 to 1p + 1 would result in a 16 % (95 % CI: 8 to 35 %) increase in infant IPD impact. Following availability of additional data, these models will be refit to the Gambia and Fiji trial data. IVIR-AC strongly suggests that the modeling incorporates results from ongoing clinical trials as these are made available. Additionally, IVIR-AC recommends extending the analysis to report both the epidemiological impact and cost-effectiveness of switching to a 1p + 1 dose schedule.

To assess the impact of PCV mass vaccination campaigns, the modeling team simulated a campaign across demographics of an internally displaced person (IDP) camp in Somaliland. They found that single-dose PCV campaigns were estimated to be effective and efficient in reducing pneumococcal disease in crisis-affected populations. However, broader age targeting may be needed to mitigate suboptimal impact in settings with a high degree of mixing with an unvaccinated population, or high migration rates. A single-dose PCV campaign among under 5-year-olds was conducted in January 2023 in Somaliland using the 10-valent PCV, Pneumosil; its impact in subsequent years will be evaluated in 2025. In the context of limited available data for modeling mass vaccination campaign scenarios in humanitarian crisis settings, IVIR-AC recommends quantifying the main drivers of estimated differences in vaccine campaign impact in different displaced population settings. In Niger, a trial was conducted to assess the impact of a mass campaign using a single PCV fractional dose (1/5 of a full dose) of Pneumosil. Estimated vaccine effectiveness against pneumococcal colonization of the campaign dose was 43 % (95 % CI: 35–48 %) for the fractional dose arm and 54 % (95 % CI: 47–61 %) in the full dose arm. Overall, analyses suggested fractional dose campaigns were as effective as a booster and may reduce costs (i.e., have a lower cost-to-benefit ratio) than a full dose campaign. Given the current uncertainty regarding fractional booster dose efficacy, IVIR-AC recommends testing the sensitivity of model outputs to this assumption. Finally, across all modes of PCV delivery modeled, IVIR-AC strongly recommends that the proposed work plan be updated to describe how results of modeling for each of the multiple trials will be compared and synthesized to support revised vaccination strategy design by the SAGE working group.

### 3.2 Session 2: Vaccine impact modeling for chikungunya

Chikungunya is a growing public health concern as the number and size of outbreaks, as well as their geographic distribution, have increased over the past few decades. IXCHIQ, the first chikungunya vaccine, has been licensed by the U.S. Food and Drug Administration (FDA) [5] in 2023 and the European Medicines Agency (EMA) [5] in 2024 via accelerated pathways that relied entirely on evidence of immunogenicity; however, no high-burden country has yet licensed the vaccine. Due to sporadic occurrence of outbreaks without a clear periodicity in many locations, it remains unclear how best to implement vaccine delivery including in responsive (i.e., in response to an emerging outbreak) versus routine immunization programs and the role of age-specific vaccination strategies. Additionally, the burden of chikungunya, in particular long-term sequelae, remains poorly described. Given these uncertainties, it is critical to use modeling methods to compare the benefits of different vaccination strategies, especially where vaccine implementation could have the greatest and most cost-effective impact. A SAGE working group is anticipated to provide SAGE policy recommendations in 2025 for vaccine use in endemic countries.

IVIR-AC was asked to comment on the feasibility and completeness of the proposed modeling questions, which include in which epidemiological settings should the vaccine be used, the use of different implementation strategies across settings, and the target age-range for vaccines.

During the session, a team from the US Centers for Disease Control and Prevention presented data on known chikungunya morbidity and mortality (i.e., 0.01–0.5 % of cases), the risk of severe disease being concentrated in the very young and elderly, and the high morbidity in adult age groups. A team from the University of Cambridge presented evidence on how modeling may help address the key challenges of quantifying the potential impact of a chikungunya vaccine. Specifically, modeling may help estimate transmission history and quantify differences in burden by age, sex, and location. The team provided examples using a literature review of reported chikungunya cases and suitability maps of *Aedes* mosquito vectors [6] to classify countries as endemic, epidemic, or no-transmission along with evidence quality (i.e., good or poor). Additionally, the team demonstrated that seroprevalence data following a chikungunya outbreak in Paraguay could be used to estimate disease burden across sex and age groups. The team also highlighted that serologic datasets across countries could be used to fit models which estimate the duration and size of outbreaks for epidemic countries [7] and the average annual force of infection for endemic countries. Due to the limited availability of burden data, disease epidemiology, seasonality, and the often sporadic nature of chikungunya transmission, IVIR-AC notes that there are challenges in firmly characterizing settings as either “outbreak” or “endemic”, and that this characterization of a particular geographic region may change over time. IVIR-AC recommends that modeled scenarios should explore this uncertainty, for example, by applying sensitivity analyses of the criteria used for archetype classification.

Additionally, as the IXCHIQ vaccine was approved based on immunogenicity data alone, there are currently no direct estimates of vaccine efficacy against disease and clinical outcomes or duration of protection. Furthermore, data are lacking to determine whether vaccination prevents infection or only disease manifestation. The team presented evidence on reductions in disease burden under different vaccination strategies and different assumptions about the vaccine. IVIR-AC notes that chikungunya vaccine characteristics important for estimating vaccine impact are largely unknown. IVIR-AC recommends that modeling be used to explore a “minimum vaccine profile” – that is, the vaccine characteristics required for the chikungunya vaccine to achieve a desired “number needed to vaccinate” to avert one case (or disability adjusted life year (DALY)). This “minimum vaccine profile” could then be varied in order to explore uncertainties in both endemic and outbreak settings.

The team highlighted an example of data from the outbreak in Paraguay used to fit compartmental transmission models that can recover the observed number of cases and immunity in the population. Under different assumed scenarios of timing of vaccine deployment, proportion of population vaccinated, vaccine efficacy, and vaccine mode of protection, models estimated how many cases and deaths would have been averted. Overall, across simulations, vaccination appears to be effective in reducing burden, but would require stockpiling in many settings to achieve notable impact.

Given that the target vaccination group may change depending on whether the objective is to minimize overall morbidity or mortality, IVIR-AC recommends estimating DALYs to address prioritization questions. IVIR-AC notes that, while deaths due to chikungunya are low overall, mortality captured in the available surveillance data is likely higher in young children and the elderly. Additionally, IVIR-AC recommends modeling, using multiple years of historical data (including non-outbreak years), to establish a threshold to prompt a reactive vaccination campaign, accounting for outbreak detection sensitivity and specificity. Lastly, IVIR-AC notes that model scenarios assumed that periodic campaigns target only the unvaccinated population, which may overestimate the impact of vaccination, since such specific targeting is difficult to achieve in practice. IVIR-AC recommends additional modeling scenarios that incorporate heterogeneity in the existing vaccination status of cohorts targeted for vaccination.

### 3.3 Session 3: Immunization coverage estimation

Each year, WHO and UNICEF produce annual estimates of national immunization coverage for childhood vaccines, known as “WUENIC” [8]. The methodology for producing these estimates is a rule-based, data triangulation framework that has been reviewed and endorsed by external bodies, including IVIR-AC [9], and it is GATHER-compliant [10]. As more vaccines are introduced across the life span and there is increasing demand for subnational coverage estimates, the WUENIC methodology needs to adapt to this changing immunization landscape. The WUENIC team proposed to establish a vaccination coverage estimation IVIR-AC subgroup to support WHO and UNICEF by providing independent technical advice on prioritization and methodological improvements to the WUENIC estimates across various antigens. IVIR-AC was asked to comment on areas of the proposed subgroup requiring clarification, highlight any issues, and provide feedback on the proposed terms of reference (TOR) and the membership of the IVIR-AC subgroup.

During the session, members of the WUENIC team presented the current WUENIC methodology. WUENIC estimates are publicly available for 15 vaccine-doses, from 1980 to the present, and published with annual revisions. The data used throughout the WUENIC process are based on country-reported coverage data from the WHO Joint Reporting Form, surveys, published literature, and other contextual information. While there is no statistical uncertainty associated with WUENIC estimates, each estimate is given a grade of confidence [11]. Overall, IVIR-AC acknowledges the extensive modeling efforts made to improve coverage monitoring in response to a previous IVIR-AC review in March 2019 when IVIR-AC noted the need to evaluate the strengths and weaknesses of alternative methods and recommended setting up an IVIR-AC subgroup to allow continuing dialogue between IVIR-AC and the WUENIC team on the best ways to estimate vaccine coverage.

The team shared that WUENIC estimates are primarily used to assess national immunization performance trends, plan and provide a framework for goal setting, and for advocacy. However, more recent use cases have shifted to WUENIC estimates informing performance-based financing decisions, forecast and assess disease risk, and estimate sub-national vaccination coverage. The team presented current challenges including: many countries meeting high thresholds for coverage; countries with no or infrequent surveys; highly mobile populations (e.g., due to conflict); rapid demographic transition; life-course vaccination (e.g. human papillomavirus (HPV) vaccine); new vaccine introductions, including with sub-national targeting (e.g., malaria vaccines); variable schedules (e.g., multiple inactivated poliovirus vaccine (IPV) and bivalent oral poliovirus vaccine (bOPV) schedules); potential for using additional data sources only available for some countries; and the desire for sub-national coverage estimates for selected countries. IVIR-AC acknowledges the need to re-visit how vaccination coverage is measured and monitored with an increasing number of new vaccines across the life course (e.g., HPV), changing demography, and need for finer spatial resolution.

To address the emerging challenges, the WUENIC team is requesting support from an IVIR-AC subgroup on vaccination coverage estimation to provide independent technical advice on coverage and related data analysis and quality. The proposed scope of the subgroup includes providing independent technical guidance on vaccination coverage measurements and estimation methodologies; advice on effective visualization, dissemination, and guided use of estimates; prioritization of vaccines to be included in the WUENIC estimates; advice on linkages with related work by other groups; inputs to the development of a research agenda on estimation strategies; and guidance on sub-national estimation approaches and priorities.

IVIR-AC agrees with the proposed terms of reference for an IVIR-AC subgroup on vaccination coverage estimation while acknowledging that the scope of work will require further refinement and consultation between the WUENIC team and the IVIR-AC Secretariat. In the proposed scope of work, IVIR-AC supports the prioritization of sub-national vaccination coverage estimates for selected countries and the addition of new vaccines in the WUENIC estimates, recognizing the need to explore new data sources and methodological approaches. IVIR-AC recommends ensuring that composition of the IVIR-AC subgroup is multi-disciplinary and includes expertise such as demography, monitoring and evaluation, digital health, and specific vaccines as relevant (e.g., IPV, HPV, malaria). IVIR-AC recommends including at least one expert from a national EPI program in an LMIC and/or Regional Immunization Technical Advisory Group. IVIR-AC recommends close working relationships with other technical groups and stakeholders involved in vaccine coverage estimation, including data collection and quality.

### 3.4 Session 4: The Lancet Commission on strengthening the use of epidemiological modeling of emerging and pandemic infectious diseases

The Lancet Commission on Strengthening the Use of Epidemiological Modeling of Emerging and Pandemic Infectious Diseases [12] aims to establish best practices and guidelines surrounding model development, evaluation, communication, and evidence translation into policy during public health emergencies. IVIR-AC was asked to comment on the progress-to-date for the Commission's work, including: (i) the suitability of list of proposed outputs from the Commission, particularly on the reporting guideline development; and (ii) the creation of the SPOTT framework (Situation, Purpose, Question, Time Horizon, Timeliness of Modeling Work, Output Types) to facilitate engagement between policy makers and modelers on model co-creation for infectious disease modeling. Additionally, the WHO Secretariat requested feedback from IVIR-AC on how to best leverage these findings for the *WHO guidance to translate modeling to support evidence-informed decision-making for immunization* currently developed by IVIR-AC subgroup.

Research activities of the Commission include four reviews of existing guidelines and frameworks, a mixed-methods study to understand the use of models to inform policies from key stakeholders, an open call for case studies on the use of models to inform policies, and explorations of best practices from other research areas, code of conduct, and ethics of modeling. The main outputs of the Commission will be: Lancet report to be finalized by October 2025; conceptual map of archetypes of different institutional arrangements for modeling for policymaking; template for translating policy questions into research questions (SPOTT); taxonomy of infectious disease models; reporting guidelines for modeling reports; communication tools and guidelines; strategies for resource mobilization to increase modeling capacity; omnibus paper discussing case studies on models that have informed policymaking; and code of conduct for modelers.

Overall, IVIR-AC welcomes the establishment of the Lancet Commission. IVIR-AC agrees that the proposed outputs from the Commission are appropriate and offers a mid-term review as the Commission's work proceeds. IVIR-AC envisages that the Commission's output will be applicable to and should be used by the wider modeling community for work on all vaccine-preventable diseases. In addition, IVIR-AC recommends that the Commission's output provide guidance on: (i) proposed strategies for low- and middle-income countries (LMICs); (ii) data quality considerations; (iii) how local context influences modeling questions, strategy, and interpretation; and (iv) ethics and other considerations regarding collaboration between commissioning agencies and modelers, including those between LMICs and high-income countries (HICs). Overall, IVIR-AC notes that there are synergies between the work of its subgroup developing the ‘*WHO Guidance to translate modelling to support evidence-informed decision-making for immunization*’ document and of the Lancet Commission and encourages the integration of their outputs.

A member of the Commission team provided information on the SPOTT framework, a communication tool supporting policymakers or scientific advisors that are commissioning infectious disease modeling studies. IVIR-AC supports the use of the SPOTT framework to facilitate communication between those commissioning modeling and modelers. However, given that modeling questions are difficult to formulate at the outset, IVIR-AC recommends adapting the SPOTT framework to guide collaborative ‘co-creation’ and to facilitate joint discussion between the those commissioning modeling and modelers throughout the modeling process. Furthermore, IVIR-AC recommends that the framework should address items on project governance, including ownership of data, models, and results, authorship, and publication policies. Lastly, IVIR-AC recommends that the Commission formulate a methodology to pilot the SPOTT tool.

### 3.5 Session 5: Setting research priorities in immunization program strengthening for South-East Asia

An evolving landscape of immunization, marked by diverse contextual factors and emerging challenges, necessitates a dynamic and context-informed immunization-related research (IR) agenda. The WHO South-East Asian Regional Office (SEARO) is undertaking a rapid systematic IR priority-setting exercise to generate a list of priority research areas for the Region, its member states, and potential funders. IVIR-AC was asked to comment on alignment of regional with overall global IR priorities and whether the priorities support other WHO regions in their IR areas of work, on the future dissemination of the IR agenda in the region, and what IVIR-AC advice would be towards the promotion and link-up of regional IR agendas with global technical partners and funders.

During the session, two members from WHO SEARO presented ongoing work, context, and priorities of the region. Regional strategic priority objectives include: (i) establishing and strengthening capacity to identify priorities for, generate and manage innovation in the space of implementation research; and (ii) evaluating promising innovations and scale-up interventions based on available evidence. Both objectives share key focus areas on needs-based innovations and evidence for implementation. IR was highlighted as having increasing importance in the region. Across WHO SEAR countries, introductions of 23 new vaccines have been implemented since 2020, six additional vaccine introductions are planned, and nine additional vaccine introductions are under consideration. Yet, since many children remain zero-dose or partially vaccinated in the region and the presence of additional risk factors are increasing (e.g., climate change, vaccination hesitancy), vaccine-preventable disease outbreaks are emerging. Current IR in the region varies across country of origin, type of vaccine (e.g., COVID-19, measles), and across research areas (e.g., research and development, social determinants). As such, WHO SEAR deemed it critical to set priorities driven by countries in the region. This prioritization will allow for better allocation of resources, fostering collaboration among stakeholders, addressing critical needs, maximizing impact, and strategic planning across WHO SEARO, researchers and funders. WHO SEARO has embarked on an exercise to identify areas for IR prioritization. Overall, IVIR-AC acknowledges the effort undertaken by WHO SEARO and supports the outcomes they may achieve through their prioritization exercise.

For this prioritization exercise, WHO SEARO has leveraged the Child Health and Nutrition Research Initiative (CHNRI) framework [13] to identify and prioritize research questions following consultation with stakeholders and experts from the region. Following consolidation of research areas via score weighting, preliminary prioritized research areas have been identified. While this exercise is still ongoing, the following thematic areas were identified as current top priorities: adult and life-course vaccination; vaccine acceptance; data reporting, quality, and decision making; strengthening cold chain infrastructure; immunization data quality; financing for improved surveillance and data reporting; disease epidemiology data for new vaccine introduction (e.g., dengue, malaria); new technology and tools for disease surveillance and immunization data reporting; investment case models for new vaccines (e.g., HPV, typhoid); and evaluation of disease surveillance practices. IVIR-AC agrees that the identified priority IR areas align with the priorities of the Immunization Agenda 2030 [14] and thus may also be broadly applicable to other WHO regions. IVIR-AC recommends that the broad list of IR areas may be refined to identify priority questions that would result in the most impactful outcomes for member states and support a better use of resources for self-sustainability. Acknowledging that the process of prioritizing research areas is still ongoing, IVIR-AC recommends that further recruitment of experts should ensure diversity among member states and roles in immunization programs. To ensure transparency, IVIR-AC recommends a methodology annexure to the report to provide detailed descriptions of assumptions, weighting, and validation.

Following this initial exercise, all prioritized areas will be developed further into IR questions and national priorities by member states. This prioritization list has been proposed to be dynamic to allow for new and upcoming priorities to be added. Overall, this prioritization will be used as a catalyst to promote a shared research agenda and undertake impactful research in WHO SEAR. Following the member state-level prioritization, IVIR-AC notes that a mapping exercise is needed to establish the past and ongoing research and current research capacity in the region to determine gaps across the highest priority questions. This may lead to shared learnings between member states and support further dissemination and use of the IR agenda. IVIR-AC recommends creating a platform for a shared learning agenda across priority areas to support member states and link capacity within the region; however, this would require commitment across partner organizations, including financial support.

### 3.6 Session 6: Determining adequate schedules for typhoid conjugate vaccines

Typhoid fever is an important public health problem in many LMICs. Global estimates of typhoid fever burden range between 11 and 21 million cases [15]. In 2018, WHO issued a position paper on typhoid vaccination [16] recommending a single dose of typhoid conjugate vaccine (TCV) in children aged 6 months in endemic regions. Routine immunization has been introduced in six countries, and three different TCVs have been licensed and WHO pre-qualified. Recent evidence, however, has shown potentially waning effectiveness four to five years after vaccination, particularly among children vaccinated below the age of two years, in a high transmission setting. To provide timely guidance for typhoid-endemic countries, SAGE has initiated a working group [17] to analyze the evidence and determine if recommendations for TCV use need to be updated. To support SAGE, modeling work is being commissioned by WHO to provide additional insight on optimal scheduling and the potential need for a booster dose that considers age-specific incidence, setting-dependent evidence on waning effectiveness, feasibility for implementation, and cost-effectiveness; this work will conclude in mid-2025, with an expected revised WHO position paper on typhoid by the end of 2025. IVIR-AC was requested to provide its views on the scientific soundness and technical feasibility of the draft modeling questions. At a future session, IVIR-AC will be requested to review results of the forthcoming modeling work. Specifically, IVIR-AC was asked to provide feedback on whether the modeling questions were sufficiently clear and precise for the modeling to be carried out, and to suggest improvements.

Presentation during the session highlighted the SAGE working group's main objective to review evidence for SAGE to issue recommendations for updated programmatic recommendations on the use of TCV, and optimization of the current vaccination schedule, including the need for a booster. Presented results from clinical trials conducted by TyVAC, the Typhoid Vaccine Acceleration Consortium, across Bangladesh [18], Malawi [19] and Nepal [20] suggested two-year vaccine protection following a single TCV dose is estimated between 79 and 85 %. Immunogenicity kinetics vary across study settings and designs.

For commissioned modeling work, proposed scenarios for settings with medium, high, and very-high (age-specific) typhoid incidence include the following: no vaccination; a single dose of routine vaccination at nine (or 15) months, two years, or five years; routine vaccination at nine (or 15) months and a booster dose at five years; routine vaccination at nine (or 15) months and booster doses at five and 10 years; and routine vaccination at nine (or 15) months and booster doses at five and 10 years with and without catch-up campaigns to 15 years.

Overall, IVIR-AC agrees that the proposed scenarios are appropriate, accounting for the age-specific and region-specific variations in typhoid incidence and severity, and recommends using several models, if feasible, to assess robustness of results to variations in the model structure. In addition to the scenarios above, IVIR-AC recommends including vaccination scenarios targeting different age groups and comparing models with only direct effects to those incorporating both direct and indirect effects. These model comparisons should consider both short-term (i.e., following introduction in 2025) and long-term (i.e., through 2035) time horizons. Across scenarios, IVIR-AC also recommends that the following outputs be modeled and evaluated: hospitalizations, deaths, the number of TCV doses needed to prevent one typhoid case (i.e., number needed to vaccinate), and DALYs.  .

Proposed scenarios also assume coverage at nine or 15 months based on first- and second-dose measles-containing vaccine (MCV1 and MCV2) coverage; additionally, scenarios assume a coverage range of a TCV dose given at two years (e.g., 50–90 % of MCV2) and coverage at five years to be uncertain and based on school attendance. Lastly, proposed modeling scenarios include two vaccine efficacy waning scenarios be to Bangladesh-like (i.e., faster) and Malawi-like (i.e., slower). IVIR-AC notes that modeling results to inform optimal schedules may be impeded by uncertainties and limitations in the vaccine efficacy waning evidence from the Bangladesh and Malawi studies. IVIR-AC supports continued collection of data to generate additional evidence and real-time data sharing with modeling teams to reduce uncertainty. IVIR-AC notes that it is important for modelers to specify assumptions regarding the target age of immunization related to immunogenicity and vaccination coverage. Lastly, IVIR-AC recommends that modeling scenarios should also consider primary and booster vaccinations (including various schedule and booster efficacy assumptions) in Bangladesh-like (i.e., high incidence) archetypes with within-setting heterogeneity.

## 4 Recurring themes

IVIR-AC continues to emphasize the importance of access to available data and updating models to reflect the most recent and highest quality data as they become available. When data are not available, across sessions, full explorations of uncertainty using alternative assumptions and sensitivity analyses should be used. Additionally, IVIR-AC looks forward to ongoing engagement and synergies between various IVIR-AC subgroups and WHO and regional initiatives to improve immunization-related research.

IVIR-AC will next convene from 17th – 21st February 2025.

## WHO author disclaimer

The authors have no interests to declare. Philipp Lambach and Mitsuki Koh work for the World Health Organization (WHO). The authors alone are responsible for the views expressed in this publication and they do not necessarily represent the decisions, policy or views of the WHO.

## CRediT authorship contribution statement

**Philipp Lambach:** Project administration, Writing – review & editing. **Sheetal Silal:** Investigation, Writing – review & editing. **Alyssa N. Sbarra:** Conceptualization, Writing – original draft. **Mitsuki Koh:** Project administration, Writing – review & editing. **Rakesh Aggarwal:** Investigation, Writing – review & editing. **Habib Hasan Farooqui:** Investigation, Writing – review & editing. **Stefan Flasche:** Investigation, Writing – review & editing. **Alexandra B. Hogan:** Investigation, Writing – review & editing. **Sun-Young Kim:** Investigation, Writing – review & editing. **Kathy Leung:** Investigation, Writing – review & editing. **William J. Moss:** Investigation, Writing – review & editing. **Patrick K. Munywoki:** Investigation, Writing – review & editing. **Allison Portnoy:** Investigation, Writing – review & editing. **Meru Sheel:** Investigation, Writing – review & editing. **Xuan-Yi Wang:** Investigation, Writing – review & editing.

## Declaration of competing interest

P. L. is supported financially by the Bill & Melinda Gates Foundation. S. S. was supported by the World Health Organization for this work. A. N. S. was financially supported by the World Health Organization for this work, and is additionally supported by the Bill & Melinda Gates Foundation, Gavi, the Vaccine Alliance, and the National Institutes of Health. M. K. is supported by the Bill & Melinda Gates Foundation. A. B. H. was supported by the Australian National Health and Medical Research Council for this work, is additionally supported by PATH, the World Health Organization, and Gavi, the Vaccine Alliance, and has received consulting fees from the Australian NSW Ministry of Health, WHO Europe and Asian Development Bank. A. P. is supported by Gavi, the Vaccine Alliance, Imperial College London, the Bill & Melinda Gates Foundation, and the World Health Organization. A. N. S. and A. B. H. report travel related support from the World Health Organization to attend previous IVIR-AC meetings. All other authors have no declarations.

## Data Availability

No data was used for the research described in the article.
